# In vitro anti-inflammatory and wound healing properties of *Andrographis echioides* and Andrographis paniculata

**DOI:** 10.6026/97320630018331

**Published:** 2022-04-30

**Authors:** Kaviya Selvaraj, R Gayatri Devi, J Selvaraj, A Jothi Priya

**Affiliations:** 1 Saveetha Dental College & Hospitals, Saveetha Institute of Medical and Technical Science (SIMATS), Saveetha University, Chennai 600077, India

**Keywords:** *Andrographis echioides*, Andrographis paniculata, anti-inflammatory, antioxidant, wound healing property, innovative technology

## Abstract

*Andrographis echioides* (L.) is an annual herbaceous plant in the family Acanthaceae. Anti-inflammatory is the property of a substance or treatment that reduces inflammation or swelling. Antioxidants are substances that can prevent or slow damage to cells caused by free radicals, unstable molecules that the body produces as a reaction to environmental and other pressures. They are sometimes called "free-radical scavengers. Therefore, it is of interest to analyse the anti-inflammatory and antioxidant properties of *Andrographis echioides* and Andrographis paniculata. Protease inhibitor activity was done by bovine serum albumin was added to 100µl of plant sample with increase in concentrations (100-500µg/ml). Invitro antioxidant activity was done by DPPH free radical scavenging assay. 200 µL of 0.1 mM DPPH prepared in methanol was added to 100 µL of the plant extract with increase in concentration. Based on the results from the present study, it can be concluded that *A.echioides* is found to be a good natural antioxidant source and *A. paniculata* is found to be a good anti-inflammatory source. However, both the plant *A.echioides* and A.paniculata have these properties. Data shows that both *A.echioides* and *A. paniculata* have potential anti-inflammatory and antioxidant activity which could be due to the presence of bioactive compounds present in the plant extracts.

## Background:

Inflammation is the defensive mechanism of tissue to any injury which may be caused by injection of chemical/physical agents but may lead to development of inflammatory bowel disease 1]. It leads to pain, heat, redness, swelling and loss of function of affected parts. Various therapeutic approaches are available for reducing long term inflammatory response. These anti-inflammatory agents exert various effects that result in reduction in the number and activity of immune cells. Several natural products are being used as good anti-inflammatory agents without the risk of side effects from the time immemorial. Antioxidants act as a major defense against radical mediated toxicity by protecting the damage caused by the free radicals. Potential antioxidant therapy should therefore include either natural antioxidant enzymes or agents which are capable of augmenting the function of these oxidative free radical scavenging enzymes [2]. Anti inflammatory is a substance that reduces swelling and inflammation [3]. The antioxidant capacity and total content of the phenolic compounds in ethanolic extracts of leaves, stems and fruits of the plant was studied. It was evaluated that free radical scavenging and superoxide dismutase activities have more content in the leaf extracts, that the highest antioxidant potential followed by stems and fruits. A positive correlation between the free radical scavenging capacity and the content of phenolic compounds was found in leaf, stems and fruits. Antioxidants significantly delay and prevent the oxidation process with low concentration and play a major role in lung and liver infection [4]. *A. paniculata* is commonly known as Nilavembu and it is also known as green chirerrata. The occurrence of paniculata is more in Sri Lanka and widely distributed in plains and hilly areas. *A. paniculata* is rich in antidiabetic, anti-inflammatory and antioxidant properties. It is an ayurvedic and traditional Chinese medicine for treating various ailments, including those of the liver [5]. *A.echioides* is also known as false willow which is a dicotyledon plant. It is closely related to *A. paniculata*. It is native to Asia and endemic in India. Herbs and leaf of *A.echioides* is used in siddha, Ayurveda for its antiulcer, antioxidant and anti inflammatory activity. *A.echioides* has importance in its leaf which cures fever, goiter, liver diseases and fungal diseases. Boiling of these leaves decreases falling and graying of hair. The antioxidant activities of the plant found that methanol extract exhibits free radical scavenging activity better than other extracts. The methanol extract exhibited the highest DPPH scavenging activity that exhibited best antioxidant activity with a value of 51.98 mg/ml [6 - check with author]. We have document several data related to the use of medical plant extracts in the potential combat of several diseases [7-26]. Therefore, it is of interest to evaluate the anti-inflammatory and wound healing properties of *A.echioides* and *A. paniculata*.

## Materials & Methods:

### Chemicals:

All chemicals and reagents used for this research work were purchased from Sigma Chemical Company St. Louis, MO, USA; Invitrogen, USA; Eurofins Genomics India Pvt Ltd, Bangalore, India; New England Biolabs (NEB), USA

### Collection of plant material:

A echioides and A paniculata were collected from Chennai District, Tamil Nadu, India. The species were identified and authenticated at the Department of Centre for Advanced Study in Botany, University of Madras, Chennai, India. The bark, leaves and flower parts of the plant were shade-dried, cut into small pieces and coarsely powdered. The coarse powder was used for extraction with ethanol.

### Preparation of plant extracts:

The 1kg of dry powders from leaves from both plants was taken in individual aspirator bottles; 3 liters of ethanol was used and the mixture was shaken occasionally for 72 hours. Then the extract was filtered. This procedure was repeated three times and all extracts were decanted and pooled. The extracts were filtered before drying using whatman filter paper no 2 on a Buchner funnel and the solvent was removed by vacuum distillation in a rotary evaporator at 40°C; the extracts were placed in pre-weighed flasks before drying.

### Assessment of in vitro anti-inflammatory activity by plant extract:

Inhibition of albumin denaturation:

The anti-inflammatory activity of the plant extract was studied by the inhibition of albumin denaturation technique which was studied according to the methods of [27] followed with minor modifications. The reaction mixture consisted of test extracts and 1% aqueous solution of bovine albumin fraction, pH of the reaction mixture was adjusted using a small amount of 1N HCl. The plant extract with increase in concentration (100 to 500 µg/ml) were incubated at 37°C for 20 min and then heated to 51°C for 20 min, after cooling the samples the turbidity was measured at 660nm.( UVVisible Spectrophotometer Model 371, Elico India Ltd) The experiment was performed in triplicate. In this study, Aspirin was used as a standard anti-inflammatory drug.

### Calculation:

% Inhibition=100-((A1 -A2)/A0)*100)

### Statistical analysis:

The data were analysed statistically using one way analysis of variance (ONE-WAY ANOVA). Duncan Multiple range test was used to analyze the statistical significance between groups. The levels of significance were considered at the levels of p<0.05.

## Results:

### Antiinflammatory activity of *A.echioides*:

In the present study, *A.echioides* significantly (p<0.05) inhibited the protein denaturation in a dose-dependent manner (100-500µg/ml). However, 400 and 500µg concentrations exhibited the maximum activity in inhibiting the denaturation of albumin protein (Figure 1 - see PDF).

### Antiinflammatory activity of *A. paniculata*:

In the present study, *A. paniculata* significantly (p<0.05) inhibited the protein denaturation in a dose-dependent manner (100-500µg/ml). However, 400 and 500µg concentrations exhibited the maximum activity in inhibiting the denaturation of albumin protein (Figure 2 - see PDF).

### In vitro antioxidant activity by plant extracts:

#### Antioxidant potential of *Andrographis echioides*:

In the present study, *A.echioides* significantly (p<0.05) inhibited the DPPH radical scavenging activity in a dose-dependent manner (100-500µg/ml). However, 400 and 500µg concentrations exhibited the maximum activity in inhibiting the DPPH radical formation (Figure 3 - see PDF), suggesting that the plant has potential antioxidant activity.

#### Antioxidant potential of Andrographis paniculata:

In the present study, Andrographis paniculata significantly (p<0.05) inhibited the DPPH radical scavenging activity in a dose-dependent manner (100-500µg/ml). However, 400 and 500µg concentrations exhibited the maximum activity in inhibiting the DPPH radical formation (Figure 4 - see PDF), suggesting that the plant has potential antioxidant activity.

## Discussion:

The protein denaturation inhibitory activity of *A.echioides* extract is found to be, at 100µg/ml and 200µg/ml the anti inflammatory activity of the plant extract is same but, as the concentration of the standard drug increased from 300µg/ml to 500µg/ml there is a significant decrease in the plant extract of 10% (Figure 1 - see PDF). The protein denaturation inhibitory activity of *A. paniculata* extract is found to be, at 100µg/ml and 200µg/ml the anti inflammatory activity of the plant extract is same but, as the concentration of the standard drug increased from 300µg/ml to 500µg/ml there is a significant decrease in the plant extract of 10% (Figure 2 - see PDF). The protein denaturation inhibitory activity of *A.echioides* is found to be, at 100µg/ml and 200µg/ml the DPPH radical scavenging activity of the plant extract is same but, as the concentration of the standard drug increased from 300µg/ml to 500µg/ml there is a significant decrease in the plant extract of 10% (Figure 3 - see PDF). The protein denaturation inhibitory activity of *A. paniculata* is found to be, at 100µg/ml and 200µg/ml the DPPH radical scavenging activity of the plant extract is same but, as the concentration of the standard drug increased from 300µg/ml to 500µg/ml there is a significant decrease in the plant extract of 10% (Figure 4 - see PDF). These results emphasise that both the plant extracts have both anti-inflammatory and antioxidant properties. Plants have a long history of use in traditional medicine and represent a nearly limitless source of pharmaceutical compounds. In fact, plants have a large number of bioactive compounds, predominantly secondary metabolites, which are beneficial to humans [28]. Since free radicals are involved in a variety of pathological events, free radical scavenging activity has a great role in normal biological function. Due to this high reactivity, the Reactive Oxygen Species (ROS) readily combine and oxidize biomolecules such as carbohydrates, proteins and lipids thus making them active with subsequent damage to cells, tissues and organs [29]. The *A. paniculata* could inhibit the superoxide radical formation in the in vitro as well as in vivo system using the rat model. This indicates that the extract possesses free radical scavenging activity of superoxide radicals and regulates the metabolites which are capable of signalling and communicating important information to the cell genetic machinery. Since the plant extract possesses free radical scavenging activity *A. paniculata* acts as a good anti oxidant agent. The *A. paniculata* extract has shown almost complete inhibition of inflammation induced by carrageenan [30]. Inflammation is mainly caused by the generation of free radicals and several reports point out the involvement of free radicals in the process of inflammation [31]. This suggests that administration of antioxidants may have a protective role in inflammatory conditions. Suppression of inflammation by *A. paniculata* extract treatment is explained by its antioxidant property and it increases the therapeutic value of the plant. These plant extracts contain anti microbial activity to which the wound healing property of the plant can be associated. Wound healing mechanisms may be contributed to stimulate the production of antioxidants in wound sites to provide a favourable environment for tissue healing which was supported by the present study [32]. The enzymatic and non enzymatic antioxidants in *A.echioides* exhibit that they possess preventive and protective roles to maintain the cell survival, cellular interaction and maintenance of cell membrane [33]. Further investigation is needed in order to fully elucidate the mechanism of *Andrographis echioides*, which have effective and therapeutic antioxidant potential against various inflammatory diseases.

## Conclusion:

Data show that both the *A.echioides* and *A. paniculata* have potential anti-inflammatory and antioxidant activity due to the presence of bioactive compounds present in the plant extracts. Hence, it is implied that *A.echioides* and *A. paniculata* can be considered as therapeutic agent. Further studies on the effect of these plant extracts on in-vivo experimental models are warranted in order to ascertain their potential action of the drugs.
